# Maternal health care utilization following the implementation of the free maternal health care policy in Ghana: analysis of Ghana demographic and health surveys 2008–2014

**DOI:** 10.1186/s12913-024-10661-5

**Published:** 2024-02-15

**Authors:** John Azaare, Gifty Apiung Aninanya, Kasim Abdulai, Francis Adane, Robert Bagngmen Bio, Martin Hushie

**Affiliations:** 1 Department of Health Service, Policy Planning, Management and Economics, School of Public Health, University for Development Studies, Tamale, Ghana; 2 Translational Nutrition Research Group, Department of Clinical Nutrition and Dietetics, University of Cape Coast, Cape Coast, Ghana; 3 Department of Health Policy, Planning and Management, School of Public Health, University of Ghana, Legon, Accra Ghana; 4 College of Health and Well-being, Kintampo, Ghana

**Keywords:** Free maternal health care policy, Antenatal care uptake, Facility delivery, Maternal health care utilization, Impact evaluation

## Abstract

**Background:**

In July 2008, Ghana introduced a ‘free’ maternal health care policy (FMHCP) through the national health insurance scheme (NHIS) to provide comprehensive antenatal, delivery and post-natal care services to pregnant women. In this study, we evaluated the ‘free’ policy impact on antenatal care uptake and facility-level delivery utilization since the policy inception.

**Methods:**

The study used two rounds of repeated cross-sectional data from the Ghana Demographic and Health Survey (GDHS, 2008–2014) and constructed exposure variable of the FMHCP using mothers’ national health insurance status as a proxy variable and another group of mothers who did not subscribe to the policy. We then generated the propensity scores of the two groups, ex-post, and matched them to determine the impact of the ‘free’ maternal health care policy as an intervention on antenatal care uptake and facility-level delivery utilization, using probit and logit models.

**Results:**

Antenatal care uptake and facility-level delivery utilization increased by 8 and 13 percentage points difference, observed coefficients; 0.08; CI: 95% [0.06–0.10]; *p* < 0.001 and 0.13; CI: 95% [0.11–0.15], *p* < 0.001, respectively. Pregnant women were 1.97 times more likely to make four plus [a WHO recommended number of visits at the time] antenatal care visits and 1.87 times more likely to give birth in a health care facility of any level in Ghana between 2008 and 2104; aOR = 1.97; CI: 95% [1.61–2.4]; *p* < 0.001 and aOR = 1.87; CI: 95% [1.57–2.23]; *p* < 0.001, respectively.

**Conclusions:**

Antenatal care uptake and facility-level delivery utilization improved significantly in Ghana indicating a positive impact of the FMHCP on maternal health care utilization in Ghana since its implementation.

**Supplementary Information:**

The online version contains supplementary material available at 10.1186/s12913-024-10661-5.

## Background

Globally, an estimated 230 million pregnancies occur annually [1, 2], thence, approximately 800 women die every day due to pregnancy or childbirth complications [3, 4]. As of 2017, global maternal mortality stood at 211 per 100,000 livebirths and although this represents a 38% decline since the year 2000, it translates to a 2.9% decline per annum and is slower than necessary to meet the Sustainable Development Goal (SDG) target of 70 per 100,000 live births by the year 2030 [4].

Low-and middle-income countries (LMICs) continue to experience a higher risk of maternal mortality of which 1 in 38 pregnancies in sub-Saharan Africa end in mortality compared to 1 in 4,300 pregnancies in Europe and Central Asia [1, 4–6]. In Ghana, the maternal mortality rate (MMR) declined from 740 to 319 per 100,000 live births between 1990 and 2015 but similar to the global trend, the rate of decline is inadequate to achieve country-level targets [7–9].

Quality antenatal care (ANC) and facility-level delivery services provided by trained health personnel have been proven to positively impact maternal healthcare outcome [10–12], yet, recent literature in sub-Saharan Africa show that nearly half of all childbirth occur at home with no support from the services of trained health personnel [7, 13, 14]. In Ethiopia for example, while experienced birth attendants provided ANC for up to 71% of pregnant women, only 16% of pregnant women gave birth under the supervision of trained personnel [15, 16]. Similarly, Ghana reported 92.1% ANC coverage in 2016 according report from the Ghana Health Service (GHS) yet, only 56% percent of pregnant women gave birth under skilled supervision [13, 17, 18].

The challenges are related to country income levels and are similar to the MMR reported globally. The World Health Organization (WHO) observed that just around 60% of pregnant women in low and middle-income countries (LMICs) have access to expert delivery services compared to 99% of pregnant women in high-income countries, thus requiring concerted efforts from LMICs [4, 5, 9].

Over the years, Ghana has implemented several maternal health policies aimed at increasing access to services among pregnant women, including; full-cost recovery, popularly called “cash and carry’ from July 1985 to May 1998, antenatal care fee exemption from June 1998 to August 2003 and delivery care fee exemption policy, initially from September 2003 to March 2005 (for four most deprived regions in Ghana-Northern, Upper East, Upper West and Central) and this was later scaled-up nationally between April 2005 and June 2007 [19–21].

Due to funding constraints, the delivery care fee exemption policy which covered antenatal care, normal, assisted and surgical deliveries ended in 2007 and pregnant women’s access to maternal health care was incorporated into the National Health Insurance Scheme (NHIS) which had begun operations since 2005 [20, 22, 23]. Subsequently, it was found that pregnant women who failed to enrol on the NHIS could not benefit from the scheme package and had challenges paying fees for maternal health care services at the service delivery points thus leading to poor uptake of skilled birth attendance in particular in most public hospitals. Therefore, the government of Ghana exempted pregnant women from paying premiums to be registered under the NHIS from July 2008 onwards, popularly called the ‘free’ maternal health care policy (FMHCP).

As part of the FMHCP, pregnant women who sought maternal health services at accredited health facilities are automatically registered with the scheme (over a period ending 3 months after delivery) to receive free comprehensive services including; ANC, pregnancy-related emergency care, normal delivery care, caesarian section delivery and post-natal care to mother and baby [21–22].

Over time, studies have examined the impact of maternity fee exemption policies, especially the delivery care fee exemption policy and found limited effect on maternal deaths [23–25] although the evidence suggests that fee exemption for maternal health care was associated with increased uptake of skilled birth care among the poor [25]. While these studies were conducted in selected regions and districts, not much is known about the impact of the current ‘free’ maternal health care policy following its integration into the NHIS. More recently, a study reviewed clinical records of 21 hospitals and found that there was an increase in out-patient attendance among pregnant women, but not facility delivery utilization [26].

Although some other studies have assessed the impact of the FMHCP on infant and neonatal mortality, the results are not only limited in focus but conflicting [24, 27]. Of particular importance is the fact that few studies have explicitly examined the impact of the FMHCP on antenatal care uptake and facility-level delivery utilization, which are the ‘free’ policy’s two major intervention variables [28], thus, the current study aimed to address this gap through an analysis of nationally representative data from the Ghana demographic and health survey using two thrend (2008–2014). Precisely, we hypothesized that the FMHCP has no significant positive impact on ANC uptake and facility-level delivery utilization in Ghana.

## Methods

### Study design

This study adopted a retrospective design using nationally representative Ghana Demographic and Health Survey (GDHS) (2008–2014), and isolated two groups of women who benefited from the FMHCP as the treatment group and those who did not benefit from the'free' policy as the no-treatment group by merging the two rounds of the repeated cross-sectional survey, pre- and post-policy. We defined antenatal care uptake as pregnant women receiving antenatal care at least four times or more considered standard requirement by WHO at the time [10, 21] and also defined facility-level delivery utilization to include all childbirths conducted in a health care facility of any level in Ghana between 2008 and 2014. At the time of conducting this analysis, the Ghana 2014 DHS was the current available DHS data sets considered wide and nationally representative with comparable variables since the ‘free’ maternal health care policy inception in 2008. The most recent DHS (Ghana: Standard, 2022)was published in May 2023. However, this report and the data it contains were unavailable at the time this manuscript was created and submitted.

### Data source

The study used two

rounds of repeated cross-sectional surveys of the Ghana DHS 2008 and 2014 extracted from the website of Measure DHS upon completion of an online application process. The DHS data sets were considered appropriate as they offered baseline and end-line data on the FMCHP’s implementation and allowed for comparison. Variables *m14* and *m15* from the original data sets were used to construct two outcomes of interest: antenatal care uptake and facility-level delivery utilization, respectively.

### Exposure variable

In practice, pregnant women have access to the FMHCP through free registration with the NHIS. Hence, women registered under the scheme were used as proxy variables and classified as having subscribed to the FMHCP and a binary variable of ‘1’ and ‘0’ constructed to represent benefiting from the ‘free’ policy or otherwise, respectively.

### Dependent variable

The current analysis considered dependent variables; antenatal care uptake and facility-level delivery utilization, analyzed and reported individually.

### Independent covariate

**Fig. 1:**
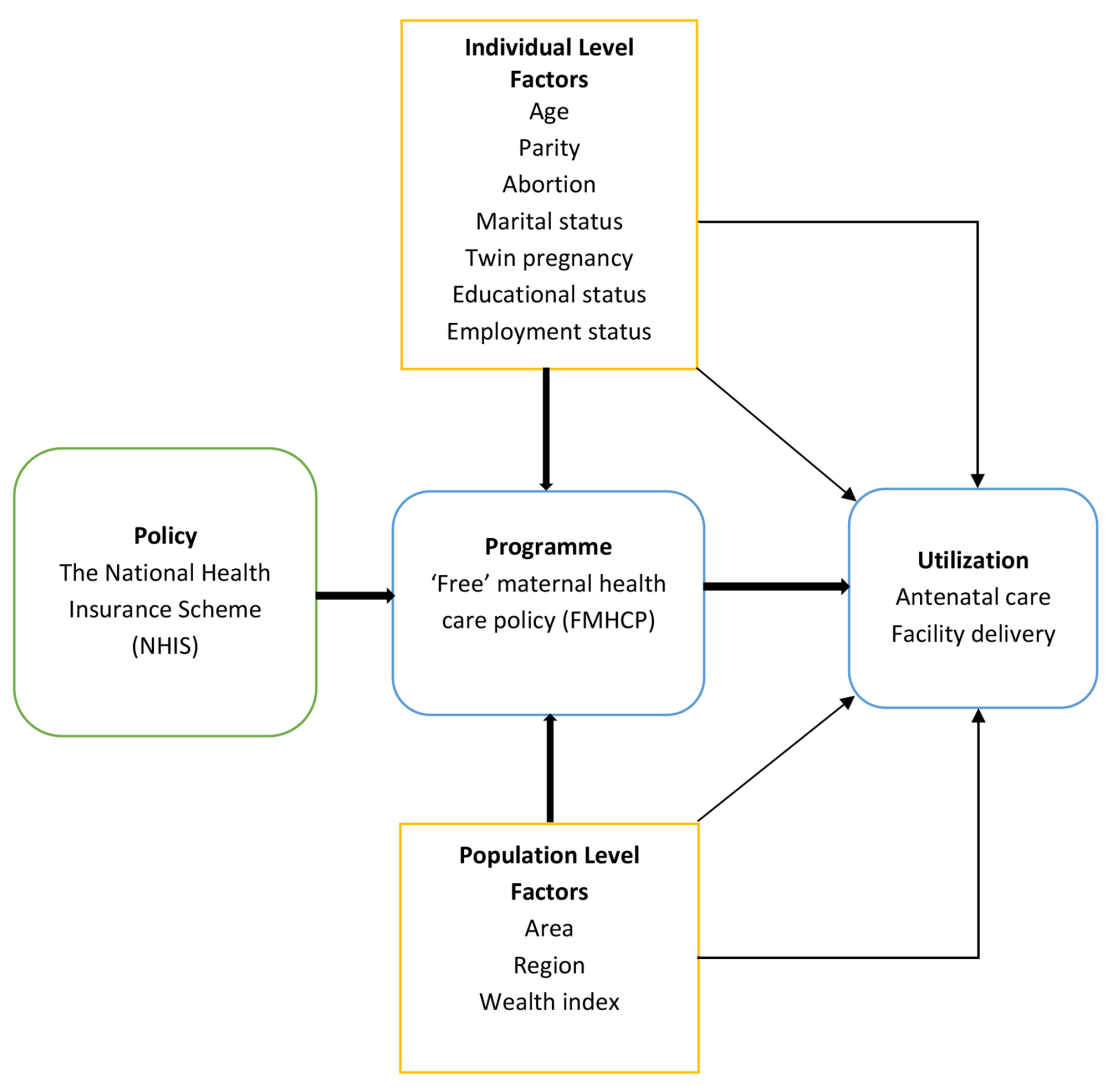
Factors influencing maternal health care utilization. (adapted from Azaare et al., 2020)

Drawing on Mosley and Chen’s conceptual framework (Fig. 1) for studying child survival rates and other literature [22], maternal age, area of residence, parity, abortion history, employment status, education, wealth index, and region were adjusted for as independent covariates for precision.

### Statistical analysis

Using STATA version 16, we constructed a binary outcome of ‘1’ representing ‘four or more antenatal care uptake’ and ‘0’ representing ‘three to zero antenatal care uptake’ as no-antenatal care uptake’. We further constructed another set of binary outcomes for facility delivery utilization, thus, ‘1’ represented ‘facility-level delivery utilization’ and ‘0’ for otherwise. The bivariate analysis of independent covariates (Table 1) was unadjusted. Results with alpha values < 0.05 were considered statistically significant and then adjusted for in the logistics regression analysis of which the results of association were reported in adjusted odd ratios (aOR). We then estimated the impact of the FMHCP as an intervention on ANC uptake and facility-level delivery utilization using the propensity scores, generated ex-post between the two groups with probit and logit models leaving the results in coefficients of determination, 95% confident interval and a statistical significance level of *p* < 0.05.

**Table 1:** Bivariate analysis of antenatal care uptake, facility delivery utilization and independent covariates

Variable	Antenatal Care Uptake			Facility Delivery Utilization		
	uOR	CI: (95%)	*P*-value	uOR	CI: 95%	*P*-value
No_FMHCP	1			1		
**FMHCP**	2.69	(2.25–3.21)	0.001***	2.37	(2.03–2.77)	0.001***
Age	1.01	(1.00-1.03)	0.006*	1.00	(0.99-1.00)	0.996
Parity	0.87	(0.84–0.90)	0.001***	0.81	(0.78–0.83)	0.001***
**History of abortion**						
No history of abortion	1			1		
History of abortion	1.59	(1.26–2.01)	0.001***	1.93	(1.62–2.31)	0.001***
**delivery place**				1		
Home delivery	1			6.03	(4.89–7.44)	0.001***
Facility delivery	1.16	(1.14–1.19)	0.001***			
**Area of residence**						
Urban	1			1		
Rural	0.34	(0.27–0.44)	0.001***	0.14	(0.11–0.18)	0.001***
**Employment**						
Unemployed	1			1		
Employed	1.25	(1.01–1.55)	0.035*	0.74	(0.60–0.91)	0.005*
**Education**						
No education	1			1		
Primary Secondary Tertiary	1.18 3.24 26.2	(0.92–1.51) (2.50–4.21) (4.59-150.16)	0.185 0.001*** 0.001***	2.01 5.69 51.6	(1.63–2.49) (4.56–7.09) (12.89–207.4)	0.001*** 0.001*** 0.001***
**Marital status**						
Unmarried	1			1		
Married Liv. tog. Widowed Divorced Not liv. tog.	1.28 0.92 0.85 1.06 1.07	(0.94–1.75) (0.66–1.28) (0.46–1.60) (0.47–2.36) (0.68–1.68)	0.107 0.624 0.632 0.876 0.767	0.64 0.65 0.39 1.21 0.75	(0.46–0.87) (0.47–0.89) (0.22–0.69) (0.58–2.53) (0.48–1.19)	0.005* 0.009* 0.001** 0.598 0.228
**Wealth Index**						
Poorest	1			1		
Poorer Middle Richer Richest	1.44 2.01 5.14 15.0	(1.11–1.88) (1.49–2.71) (3.50–7.54) (8.34–27.2)	0.006* 0.001*** 0.001*** 0.001***	2.14 4.18 13.13 39.65	(1.71–2.69) (3.21–5.43) (9.60-17.97) (25.70-61.18)	0.001*** 0.001*** 0.001*** 0.001***
**Region**						
Western	1			1		
Central G. Accra Volta Eastern Ashanti Brong-Ahafo Northern Upper East Upper West	0.91 1.56 0.44 0.44 1.43 0.96 0.35 1.13 1.15	(0.55–1.51) (0.96–2.52) (0.28–0.71) (0.29–0.67) (0.90–2.26) (0.58–1.58) (0.21–0.58) (0.67–1.93) (0.69–1.91)	0.740 0.071 0.001** 0.001*** 0.124 0.880 0.001*** 0.629 0.570	0.82 4.30 0.70 0.83 1.82 1.23 0.20 1.00 0.59	(0.57–1.18) (2.73–6.77) (0.47–1.07) (0.58–1.20) (1.26–2.64) (0.81–1.86) (0.13–0.33) (0.65–1.56) (0.37–0.96)	0.286 0.001*** 0.101 0.336 0.001** 0.321 0.001*** 0.966 0.035*

uOR - unadjusted odd ratio; 1– reference; *p < 0.05; **p = 0.001; ***p < 0.001

#### Sensitivity analysis

**Fig. 2:**
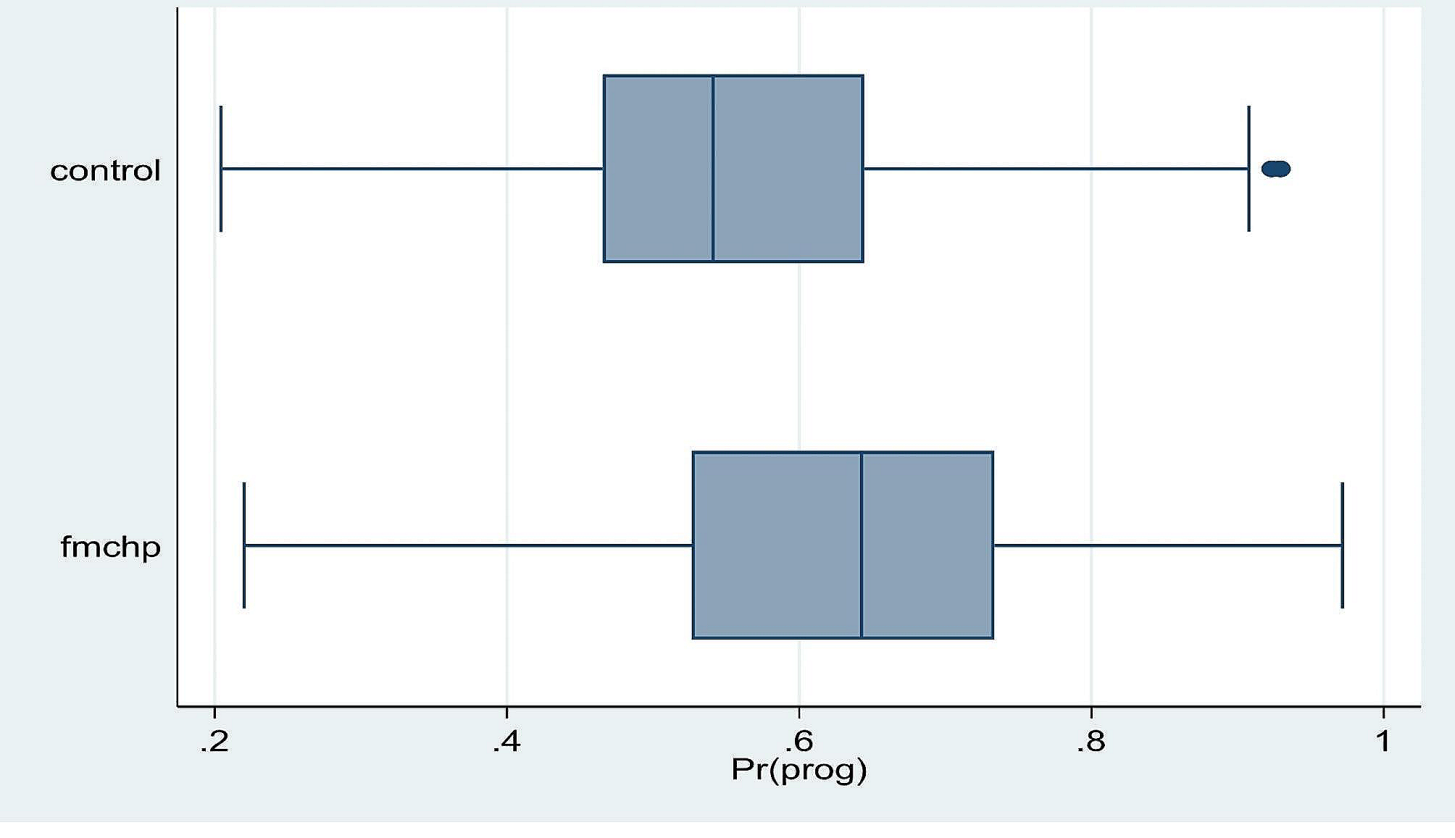
Fig. Box plot graph comparing the ‘free’ maternal health care policy and the control group

**Fig. 3:**
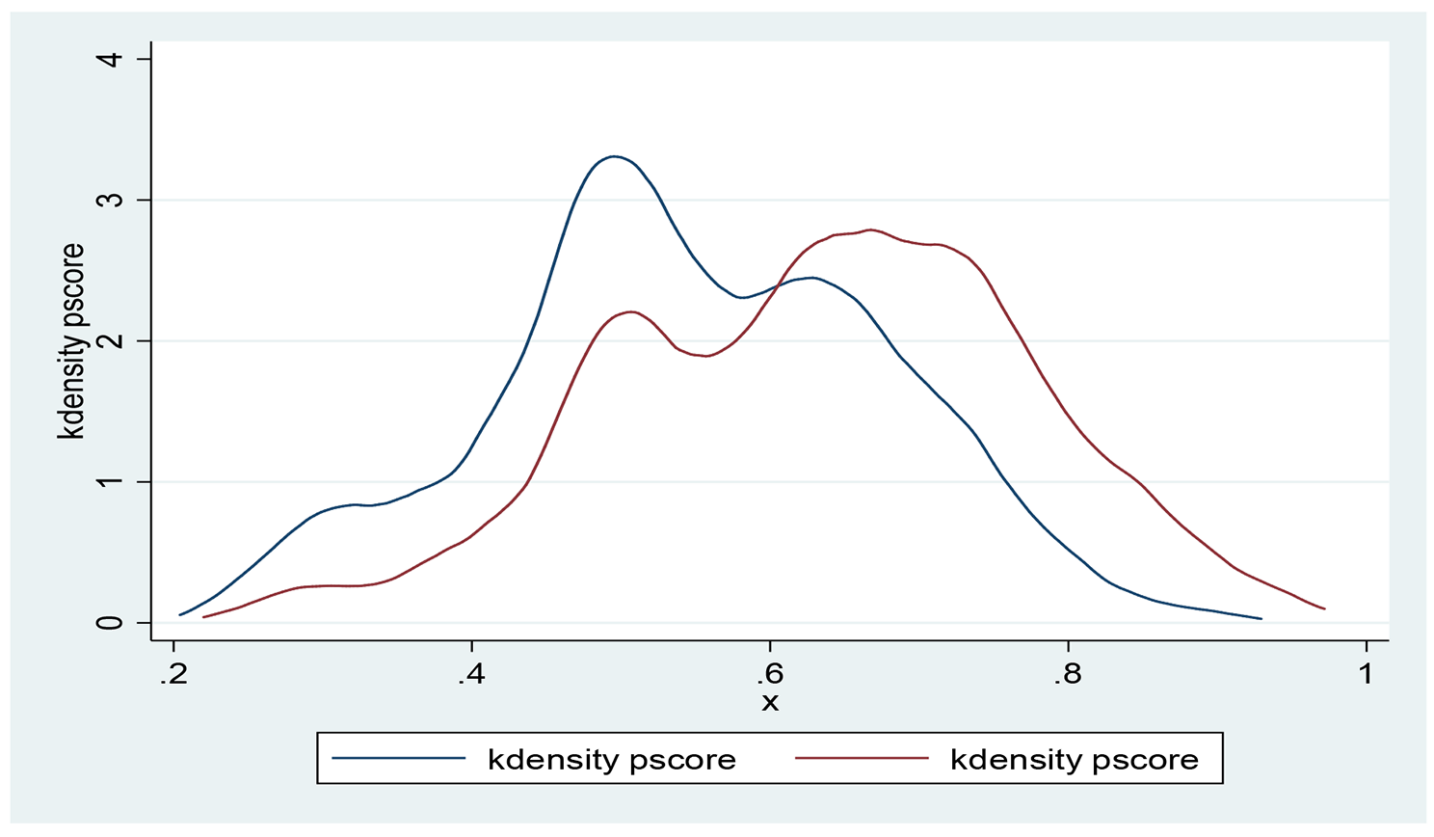
Kernel density plot showing common support for comparison between the intervention and control groups

**Fig. 4:**
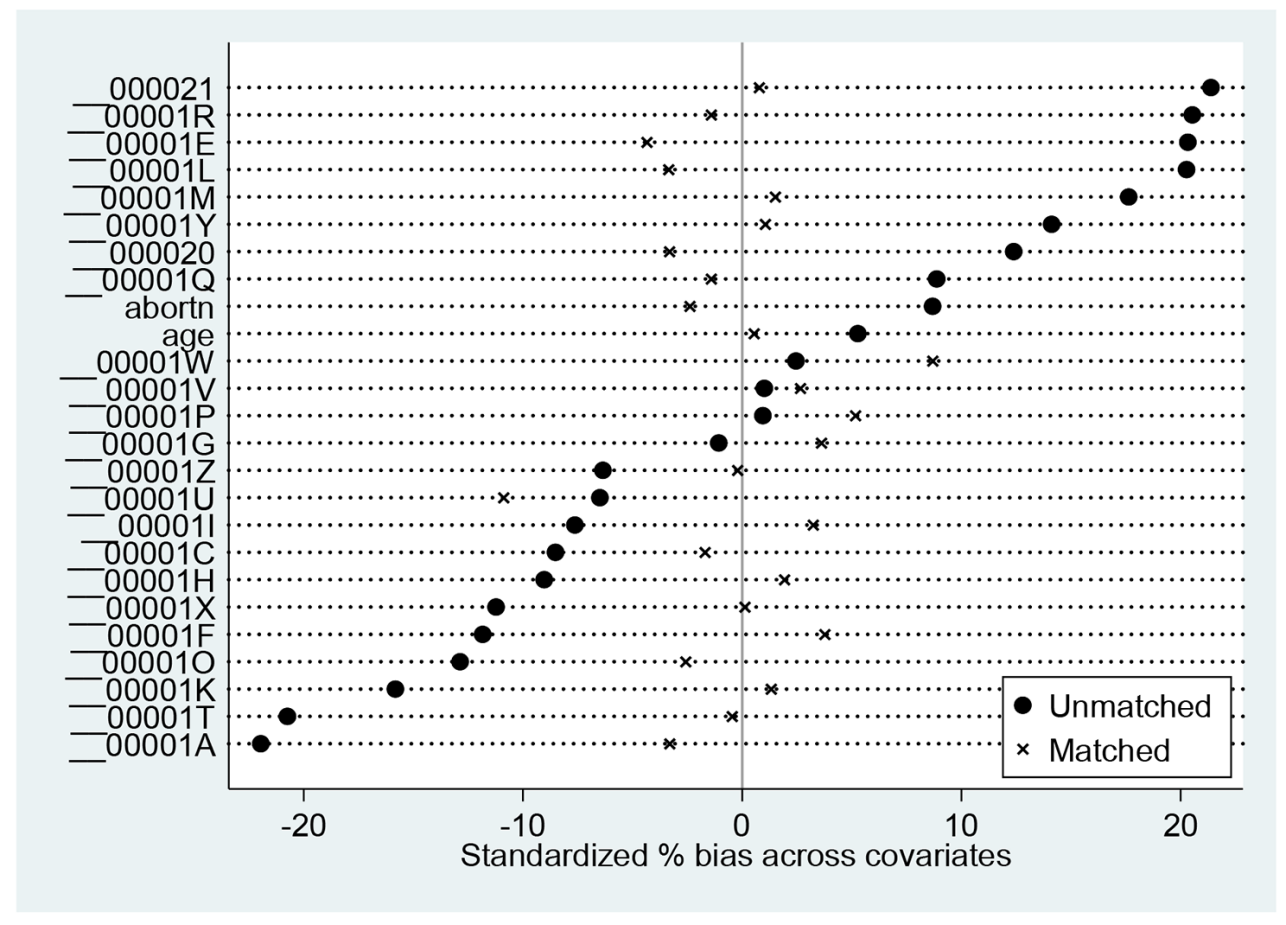
Standardized test of bias showing the matched characteristics around or close to line zero

We adjusted for the complex design of the DHS data sets by applying sample weighting using the primary sampling units (v021) and rural/urban area of residence (v022), hence selected *v005* and divided by 1,000,000 in Stata to cater for 6 decimal places (usually not accounted for in STATA version of the data sets). We then prefixed all Stata commands with *‘svy’* afterwards using Taylor linearization for reduced standard error. The models were repeated in a negative binomial regression with robust standard error to test for sensitivity and over-dispersion. Also, box plots (Fig. 2) and Kernel density curve graphs (Fig. 3) were plotted to check for overlap and common support in the matching method. We also checked for standardized bias with t-test statistics (Fig. 4).

## Results

### Descriptive statistics

Of the 8876 survey participants, the mean maternal ages were 30 and 31 years for 2008 and 2014, respectively. Mean parity and mean antenatal care were 4 and 6, respectively for both rounds of DHS data sets (Table 2) and the differences were statistically significant, *p* < 0.05 (Table 1).

**Table 2:** Descriptive statistics

Variable	Observation	2008		2014	
		n	mean	N	Mean
Maternal Age	8,876	2,992	30	5,884	31
Antenatal Care	6,360	2,088	6	4,272	6
Parity	8,876	2,992	4	5,884	4

### Associated factors of antenatal care uptake and facility delivery utilization

From Table 3, pregnant women were 1.97 times more likely to make four plus ANC visits; aOR: 1.97; CI: 95% [1.61–2.4]; *p* = 0.001. ANC uptake increased with increasing maternal age; aOR = 1.05; 95% CI: [1.03–1.07]; *p* = 0.05. Women with employment and women secondary level of education were more likely to visit ANC clinics compared to women with no employment and women with no formal education; aOR = 1.46; 95% CI: [1.16–1.84]; *p* < 0.001 and aOR = 1.59; 95% CI: [1.23–2.06]; *p* < 0.001. Women in Greater Accra; aOR = 0.54; CI: 95% [0.31–0.93]; *p* = 0.028, Volta region; aOR = 0.52; CI: 95% [0.32–0.83]; *p* = 0.007 and Eastern region; aOR = 0.44; CI: 95% [0.29–0.68]; *p* = 0.001 were less likely to make four plus antenatal care visits compared to women in the reference (Western) region.

**Table 3:** Multivariate analysis of association between the ‘free’ maternal health care policy and antenatal care uptake

Variable	Multiple logistic regression with linearized std. error			Poisson regression with linearized std. error			Negative binomial regression With linearized std. error		
	aOR	(CI: 95%)	*P*-Value	aPR	(CI: 95%)	*P*-Value	aPR	(CI: 95%)	*P*-value
No_FMHCP	Ref.			Ref.			Ref.		
**FMHCP**	1.97	(1.61–2.41)	0.001***	0.09	(0.06–0.11)	0.001***	0.09	(0.07–0.11)	0.001***
Age	1.05	(1.03–1.07)	0.001***	0.01	(0.003–0.01)	0.001***	0.01	(0.004–0.01)	0.001***
Parity	0.84	(0.79–0.90)	0.001***	-0.02	(-0.02-0.01)	0.001***	-0.02	(-0.03 -0.01)	0.001***
**Abortion**									
No history of abortion	Ref.			Ref.			Ref.		
History of abortion	1.13	(0.88–1.46)	0.312	0.01	(-0.01-0.03)	0.305	0.02	(-0.003–0.04)	0.094
**Delivery place**									
Home delivery	Ref.			Ref.			Ref.		
Facility delivery	3.20	(2.61–3.92)	0.001***	0.22	(0.18–0.26)	0.001***	0.22	(0.18–0.25)	0.001***
**Area of residence**									
Urban	Ref.			Ref.			Ref.		
Rural	1.03	(0.79–1.35)	0.774	0.01	(-0.02-0.04)	0.706	-0.002	(-0.02–0.023)	0.881
**Employment**									
Unemployed	Ref.			Ref.			Ref.		
Employed	1.46	(1.16–1.84)	0.001***	0.04	(0.007–0.07)	0.016*	0.03	(0.005 − 0.06)	0.023*
**Education**									
No education	Ref.			Ref.			Ref.		
Primary	1.02	(0.80–1.28)	0.867	-0.003	(-0.04-0.04)	0.868	0.002	(-0.03–0.03)	0.925
Secondary	1.59	(1.23–2.06)	0.001***	0.047	(0.01–0.08)	0.010*	0.04	(0.01–0.06)	0.013*
Tertiary	3.69	(0.63–21.3)	0.144	0.038	(-0.002-0.07)	0.065	0.03	(-0.01- 0.06)	0.061
**Marital status**									
Not married	Ref.			Ref.			Ref.		
Married	1.38	(0.96–1.97)	0.077	0.027	(-0.02-0.07)	0.249	0.05	(0.01–0.09)	0.028*
Liv. together	1.07	(0.73–1.56)	0.723	-0.001	(-0.05-0.05)	0.977	0.01	(-0.03–0.06)	0.624
Widowed	1.02	(0.53–1.97)	0.936	-0.001	(-0.10-0.09)	0.978	0.01	(-0.08–0.10)	0.847
Divorced	1.14	(0.50–2.59)	0.748	0.01	(-0.10-0.12)	0.880	0.01	(-0.08–0.09)	0.857
Not liv. together	1.25	(0.78–1.99)	0.343	0.02	(-0.04-0.08)	0.563	0.01	(-0.05–0.08)	0.668
**Wealth index**									
Poorest	Ref.			Ref.			Ref.		
Poorer	1.19	(0.92–1.53)	0.164	0.05	(-0.004-0.10)	0.067	0.04	(-0.001- 0.07)	0.056*
Middle	1.25	(0.92–1.71)	0.150	0.06	(0.01–0.12)	0.029*	0.06	(0.01–0.09)	0.006*
Richer	2.23	(1.42–3.49)	0.001***	0.09	(0.04–0.15)	0.001**	0.09	(0.05–0.13)	0.001***
Richest	4.72	(2.33–9.56)	0.001***	0.10	(0.04–0.16)	0.001**	0.08	(0.03–0.12)	0.001**
**Region**									
Western	Ref.			Ref.			Ref.		
Central	1.16	(0.72–1.84)	0.532	0.02	(-0.03- 0.07)	0.500	0.02	(-0.02–0.05)	0.398
G. Accra	0.54	(0.31–0.93)	0.028*	-0.04	(-0.08–0.01)	0.098	-0.02	(-0.05–0.02)	0.361
Volta	0.52	(0.32–0.83)	0.007*	-0.10	(-0.17- -0.02)	0.006	-0.09	(-0.15 - -0.04)	0.001***
Eastern	0.44	(0.29–0.68)	0.001***	-0.12	(-0.17- -0.06)	0.001***	-0.11	(-0.16 - -0.06)	0.001***
Ashanti	1.10	(0.67–1.79)	0.701	0.01	(-0.04- -0.05)	0.757	0.01	(-0.02–0.05)	0.506
Brong-Ahafo	1.03	(0.63–1.69)	0.892	0.004	(-0.05- -0.05)	0.877	0.01	(-0.02–0.05)	0.529
Northern	0.75	(0.47–1.21)	0.249	-0.05	(-0.13- -0.04)	0.253	-0.07	(-0.13 - -0.02)	0.003
Upper East	1.41	(0.80–2.49)	0.232	0.65	(0.002–0.13)	0.045*	0.06	(0.01–0.10)	0.013*
Upper West	1.67	(0.97–2.88)	0.062	0.08	(0.02–0.14)	0.011*	0.07	(0.02- 0.11)	0.005*

aCoef.– adjusted coefficient; * *p* < 0.05; **p = 0.001; ***p < 0.001

Similarly in Table 4, pregnant women were 1.87 times more likely to deliver in a healthcare facility in the FMHCP group compared to those who did not subscribe to the policy; aOR = 1.87; CI: 95% [1.57–2.23]; *p* < 0.001. Maternal age, aOR = 1.03; CI: 95% [1.01–1.05]; *p* < 0.001, secondary education, aOR = 1.78; CI: 95% [1.40–2.26], *p* < 0.001, history of abortion aOR = 1.31; CI: 95% [1.07–1.60]; *p* = 0.009, and 4 + antenatal care visits; aOR = 3.2; CI: 95% [2.61–3.92]; *p* < 0.001 predicted facility level delivery utilization and these were statistically significant in association.

**Table 4:** Multivariate analysis of association between the ‘free’ maternal health care policy and facility delivery utilization

Variable	Multiple logistic regression with linearized std. error			Poisson regression with linearized std. error			Negative binomial regression with robust std. error		
	aOR	(CI: 95%)	*P*-Value	aPR	(CI: 95%)	*P*-Value	aPR	(CI: 95%)	*P*-value
No_FMHCP	Ref.			Ref.			Ref.		
**FMHCP**	1.87	(1.57–2.23)	0.001***	0.14	(0.10–0.18)	0.001***	0.18	(0.14–0.21)	0.001***
Age	1.03	(1.01–1.05)	0.001***	0.01	(0.003–0.01)	0.001***	0.01	(0.004–0.01)	0.001***
Parity	0.87	(0.82–0.93)	0.001***	-0.03	(-0.04- -0.01)	0.001***	-0.04	(-0.05 - -0.02)	0.001***
**Abortion**									
No history of abortion	Ref.			Ref.			Ref.		
History of abortion	1.31	(1.07–1.60)	0.009*	0.05	(0.01–0.08)	0.007*	0.03	(-0.005–0.06)	0.103
**Antenatal care**									
0–3 attendance	Ref.			Ref.			Ref.		
4 + attendance	3.20	(2.61–3.92)	0.001***	0.50	(0.39–0.61)	0.001***	0.52	(0.44–0.60)	0.001***
**Area of residence**									
Urban	Ref.			Ref.			Ref.		
Rural	0.44	(0.34–0.56)	0.001***	-0.17	(-0.22- -0.15)	0.001***	-0.19	(-0.23- 0.15)	0.001***
**Employment**									
Unemployed	Ref.			Ref.			Ref.		
Employed	0.88	(0.70–1.09)	0.260	-0.04	(-0.07-0.01)	0.094	-0.03	(-0.06 − 0.004)	0.085
**Education**									
No education	Ref.			Ref.			Ref.		
Primary	1.19	(0.94–1.52)	0.142	0.08	(0.01–0.15)	0.046	0.11	(0.05–0.16)	0.001***
Secondary	1.78	(1.40–2.26)	0.001***	0.14	(0.07–0.20)	0.001***	0.17	(0.12–0.22)	0.001***
Tertiary	3.44	(0.76–15.5)	0.108	0.14	(0.6 − 0.21)	0.001***	0.16	(0.10–0.21)	0.001***
**Marital status**									
Not married	Ref.			Ref.			Ref.		
Married	0.79	(0.56–1.12)	0.197	-0.05	(-0.11-0.01)	0.099	-0.07	(-0.13- -0.24)	0.005*
Liv. together	0.79	(0.56–1.14)	0.205	-0.04	(-0.10-0.02)	0.202	-0.07	(-0.12- -0.01)	0.016*
Widowed	0.41	(0.19–0.83)	0.014*	-0.22	(-0.40- -0.04)	0.016	-0.17	(-0.32- -0.02)	0.024*
Divorced	1.87	(0.83–4.24)	0.129	0.10	(-0.03-0.24)	0.133	0.03	(-0.08- 0.14)	0.565
Not liv. together	0.79	(0.46–1.36)	0.407	-0.05	(-0.16-0.43)	0.260	-0.12	(-0.21- 0.02)	0.014*
**Wealth index**									
Poorest	Ref.			Ref.			Ref.		
Poorer	1.64	(1.34–2.01)	0.001***	0.26	(0.16–0.35)	0.001***	0.20	(0.14–0.27)	0.001***
Middle	2.08	(1.60–2.71)	0.001***	0.35	(0.25–0.46)	0.001***	0.29	(0.22–0.36)	0.001***
Richer	4.20	(2.85–6.17)	0.001***	0.43	(0.33–0.54)	0.001***	0.34	(0.27–0.41)	0.001***
Richest	6.11	(3.59–10.3)	0.001***	0.39	(0.28–0.50)	0.001***	0.31	(0.24–0.38)	0.001***
**Region**									
Western	Ref.			Ref.			Ref.		
Central	0.87	(0.55–1.37)	0.556	-0.03	(-0.13- 0.06)	0.501	0.03	(-0.04- 0.09)	0.469
G. Accra	1.55	(0.92–2.63)	0.098	0.07	(-0.01-0.15)	0.067	0.08	(0.03–0.14)	0.002*
Volta	1.21	(0.75–1.96)	0.418	0.04	(-0.06-0.16)	0.420	0.04	(-0.02- -0.12)	0.198
Eastern	1.08	(0.70–1.66)	0.707	0.02	(-0.07-0.12)	0.634	0.05	(-0.01- -0.12)	0.110
Ashanti	1.54	(0.99–2.38)	0.052	0.08	(-0.001-0.16)	0.053	0.11	(0.05–0.17)	0.001***
Brong-Ahafo	1.60	(1.00-2.56)	0.049*	0.09	(-0.001-0.19)	0.053	0.13	(0.07–0.19)	0.001***
Northern	0.62	(0.38-1.00)	0.054	-0.25	(-0.43- -0.07)	0.006	-0.21	(-0.03- -0.12)	0.001***
Upper East	2.96	(1.75–4.98)	0.001***	0.34	(0.21–0.46)	0.001***	0.32	(0.24–0.39)	0.001***
Upper West	1.45	(0.86–2.45)	0.165	0.11	(-0.03-0.26)	0.135	0.11	(0.03–0.19)	0.006*

*aPR*– adjusted Prevalence Ratio; * *p* < 0.05; *** *p* < 0.001

Pregnant women in the Upper East region, aOR = 2.96; CI: 95% [1.75–4.98]; *p* < 0.001 and Brong-Ahafo region, aOR = 1.60; CI: 95% [1.00-2.56]; *p* = 0.049 regions were more likely to give birth in a health care facility compared to pregnant women in the reference region.

### Percentage points impact of the ‘free’ policy on antenatal care uptake and facility-level delivery utilization

Antenatal care uptake and facility delivery utilization increased by 8 and 13% points, respectively, observed coefficient; 0.08; CI: 95% [0.06–0.10]; *p* < 0.001 and 0.13; CI: 95% [0.11–0.15]; *p* < 0.001, and these were statistically significant (Table 5).

**Table 5:** Impact of the ‘free’ maternal health care policy on antenatal care uptake and facility delivery utilization; kernel propensity score analysis using logit regression model

Variable	Bootstrapping standard error					
	Antenatal care uptake			Facility delivery utilization		
	ATE	(CI: 95%)	*P*-value	ATE	(CI: 95%)	*P*-value
**No**_**FMHCP**	**1**			**1**		
FMHCP	0.08	(0.06–0.10)	0.001***	0.13	(0.11–0.15)	0.001***
Age	0.003	(-0.002-0.008)	0.231	0.004	(0.0001–0.008)	0.043*
**Abortion**						
No	**1**			**1**		
Yes	0.12	(0.04–0.20)	0.004*	0.13	(0.06–0.20)	0.001***
**Area of residence**						
Urban	**1**			**1**		
Rural	-0.06	(-0.15-0.02)	0.176	-0.09	(-0.17- -0.01)	0.021*
**Employment**						
No	**1**			**1**		
Yes	-0.12	(-0.21-0.03)	0.008	-0.13	(-0.20 - -06)	0.001***
**Education**						
No education	**1**			**1**		
Primary	0.03	(-0.06-0.13)	0.486	0.06	(0.21 − 0.13)	0.151
Secondary	0.39	(0.29–0.48)	0.001***	0.41	(0.32–0.48)	0.001***
Tertiary	0.67	(0.44–0.90)	0.001***	0.68	(0.47–0.87)	0.001***
**Marital status**						
Not married	**1**			**1**		
Married	0.33	(-0.20-0.47)	0.001) ***	0.36	(0.24–0.49)	0.001***
Liv. together	0.15	(o.01-0.29)	0.035*	0.21	(0.08–0.33)	0.001**
Widowed	0.21	(-0.07- -0.49)	0.148	0.22	(-0.04-0.47)	0.103
Divorced	-0.16	(-0.44-0.12)	0.265	-0.16	(-0.42- 0.09)	0.210
Not liv. together	-0.09	(-0.11-0.31)	0.367	0.10	(-0.08- 0.29)	0.290
**Wealth index**						
Poorest	**1**			**1**		
Poorer	0.16	(0.06–0.26)	0.002*	0.16	(0.07–0.24)	0.001***
Middle	0.32	0.20–0.43)	0.001***	0.31	(0.20–0.41)	0.001***
Richer	0.43	(0.29–0.57)	0.001***	0.42	(0.29–0.54)	0.001***
Richest	0.61	(0.44–0.78)	0.001***	0.61	(0.45–0.75)	0.001***
**Region**						
Western	**1**			**1**		
Central	-0.35	(-0.49- -0.20)	0.001***	-0.40	(-0.53- -0.28)	0.001***
G. Accra	-0.49	(-0.65- -0.33)	0.001***	-0.45	(-0.58- -0.30)	0.001***
Volta	0.16	(-0.009-0.31)	0.038*	0.15	(0.01–0.28)	0.025*
Eastern	0.13	(-0.01-0.28)	0.082	0.11	(-0.02- 0.23)	0.104
Ashanti	-0.31	(-0.45- -0.17)	0.001***	-0.32	(-0.44- -0.20)	0.001***
Brong-Ahafo	0.48	(0.33–0.62)	0.001***	0.44	(0.31–0.56)	0.001***
Northern	0.34	(0.19–0.49)	0.001***	0.28	(0.15- -0.40)	0.001***
Upper East	0.61	(0.44–0.77)	0.001***	0.59	(0.44–0.72)	0.001***
Upper West	0.82	(0.65–0.99)	0.001***	0.77	(0.62–0.91)	0.001***

1– reference; aCoef.– adjusted coefficient; * Significant level < 0.05; ** Significant level = 0.001; *** Significant level < 0.001

## Discussion

The current analysis shows that pregnant women in Ghana made four or more antenatal care uptake and were more likely to deliver in a health care facility of any level from primary to tertiary service providers following the ‘free’ maternal health policy implementation and the differences are statistically significant, *p* < 0.001. With the inception of the ‘free’ maternal health care policy, pregnant women are 97% and 87% more likely to take up antenatal care services and give birth in a health care facility of any level in Ghana compared to to those in the non-free policy group.

Interestingly, while the analysis of association showed a stronger correlation between the ‘free’ maternal healthcare policy and uptake of antenatal care compared to facility delivery utilization, the policy impact estimation found an eight-percentage point difference in favour of facility-level delivery utilization in Ghana. This could be attributed to two scenarios. First, pregnant women are likely to take up antenatal care during pregnancy and only take up facility-level delivery services when complications necessitate assisted delivery and this has been reported elsewhere where pregnant women reported more complications leading to higher stillbirths in the ‘free’ policy group compared to the no ‘free’ policy group [24]. Secondly, facility delivery utilization is perhaps benefiting more from the ‘free’ policy impact relative to unconventional cost associated with delivery services uptake due to NHIS claims payment delays [25].

With the current findings, the null hypothesis is rejected as both antenatal care and facility delivery utilization benefit from the FMCHP implementation in Ghana. While lessons could be drawn from the current study, the results should be linked to specific elements of ANC uptake to determine the clinical benefits of ANC uptake in Ghana as argued by Hodgin and D’Agostino [26, 29].

Regional view of the results show that pregnant women in the Upper East Region of Ghana in particular were more likely to deliver in a health care facility compared to other regions and this evidence is similar to the findings of the ‘State of the Nation’s Health’ report by the University of Ghana School of Public Health [30]. The one main factor that could explain this is the implementation of the Community-Based Health Planning Services (CHPS)– a nationwide national health reform programme aimed at bringing health care to the doorsteps of local communities which is well embraced in the UE region compared to other regions. Secondly, as one of the poorest regions of Ghana [31], pregnant women have a higher propensity to register with the NHIS to safeguard their healthcare needs and are more likely to subscribe and use the ‘free’ compared to pregnant women in other regions.

Pregnant women in the Greater Accra, volta and eastern regions were less likely to make ANC visits between 2008 and 2014 and although the reasons are not clear within the scope of the current analysis, the odds of pregnant women paying out-of-pocket in the Greater Accra and Eastern regions are higher due to financial reasons and perceived quality of care in the private health care sector [32, 33], nevertheless, the current finding is a piece of useful evidence in setting the stage for increasing investment in maternal health care in Ghana to support the realization of the global targets of 70 per 100,000 live birth maternal mortality rate by 2030. The finds of the current study have implications for policy and practice as it outlines Ghana’s potential to achieve the WHO recommendation of eight minimum contact visits required for pregnant women to receive adequate maternity care in the country [10, 11].

### Strengths and limitations

Using two rounds of Ghana DHS data afforded the authors a large pool of nationally representative data useful for generalizations. Also, applying multiple models to arrive at a convergence aided model predictability and sensitivity and added quality to the current analysis. Nonetheless, the study has limitations as well. Propensity score matching assumes that unobserved characteristics are similar and cancel out. Although the GDHS (2008-2O14) was the most recent data available from the DHS databa, the lapse of time suggests that the study findings should be interpreted with caution.

## Conclusion

In conclusion, both antenatal care uptake and facility-level delivery utilization are benefiting from the current ‘free’ maternal health policy in Ghana, hence an opportunity to increase investment in the policy and by extension the national health insurance scheme to ensure policy continuity to maximize gains towards the achievement SDG 3 in Ghana and sub-Saharan African countries.

### Electronic supplementary material

Below is the link to the electronic supplementary material.

Supplementary Material 1

## Data Availability

The datasets generated and/or analysed during the current study are available in the DHS programme repository [https://dhsprogram.com/data/].

## Abbreviations

ANC: Antenatal care
CSD: Cesarean Section Delivery
DHS: Demographic and Health Survey
FMHCP: Free Maternal Health Care Policy
FDU: Facility Delivery Utilization
GDHS: Ghana Demographic and Health Survey
GHS: Ghana Health Service
MOH: Ministry of Health
NHIA: National Health Insurance Authority
NHIS: National Health Insurance Scheme
WHO: World Health Organization

## References

[1] Alkena L, Chou D, Hogan D, Zhang S, Moller A-B, Gemmill A et al. National, regional, and global levels and trends in maternal mortality between 1990 and 2015 with scenario-based projections to 2030: a systematic analysis by the United Nations Maternal Mortality Estimation Inter-agency Group. Lancet (London, England). 2017;387(10017):462–74. 10.1016/S0140-6736(15)00838-7 PMC5515236 26584737

[2] Dickson KS, Darteh EKM, Kyereme-Kumi A. Providers of antenatal care services in Ghana: evidence from Ghana demographic and health surveys 1988–2014. 2017;17(203):1–9. 10.1186/s12913-017-2145-z PMC5348873 28288647

[3] WHO. World Health Statistic., 2016. Geneva; 2016.

[4] UNICEF, UNFPA, UNFPA UNICEF, WBG UN. Trends in maternal mortality 2000 to 2017: estimates by WHO. World Bank Group and the United Nations Population Division, Geneva. Geneva; 2019. p. 119.

[5] UN. The Millennium Development Goals Report 2013. United Nation. New York; 2013.

[6] Banke-Thomas A, Avoka CKO, Gwacham-Anisiobi U, Omololu O, Balogun M, Wright K (2022). Travel of pregnant women in emergency situations to hospital and maternal mortality in Lagos, Nigeria: a retrospective cohort study. BMJ Glob Heal.

[7] Gudu W, Addo B. Factors associated with utilization of skilled service delivery among women in rural Northern Ghana: a cross sectional study. 2017;1–10. 10.1186/s12884-017-1344-2 PMC5452376 28566088

[8] Cofie LE, Barrington C, Singh K, Sodzi-tettey S, Akaligaung A. Birth location preferences of mothers and fathers in rural Ghana: Implications for pregnancy, labor and birth outcomes. 2015;1–8. 10.1186/s12884-015-0604-2 PMC4534058 26265087

[9] Kruk ME, Gage AD, Arsenault C, Jordan K, Leslie HH, Roder-DeWan S (2018). High-quality health systems in the Sustainable Development goals era: time for a revolution. Lancet Glob Heal.

[10] Lattof SR, Moran AC, Kidula N, Moller AB, Jayathilaka CA, Diaz T (2020). Implementation of the new WHO antenatal care model for a positive pregnancy experience: a monitoring framework. BMJ Glob Heal.

[11] Benova L, Tunçalp Ö, Moran AC, Campbell OMR (2018). Not just a number: examining coverage and content of antenatal care in low-income and middle-income countries. BMJ Glob Heal.

[12] World Health Organization. A neglected tragedy: the global burden of stillbirths. New York; 2020.

[13] Ayanore MA, Pavlova M, Groot W. Focused maternity care in Ghana: results of a cluster analysis. BMC Health Serv Res [Internet]. 2016; 10.1186/s12913-016-1654-5. 10.1186/s12913-016-1654-5 PMC4989378 27534617

[14] Ganle JK (2016). Ethnic disparities in utilisation of maternal health care services in Ghana. Ethn Heal.

[15] Fekadu M, Regassa N. Skilled delivery care service utilization in Ethiopia: analysis of rural-urban differentials based on national demographic and health survey (DHS). data. 2014;14(4). 10.4314/ahs.v14i4.29 PMC4370080 25834510

[16] Turi E, Fekadu G, Taye B, Kejela G, Desalegn M, Mosisa G (2020). The impact of antenatal care on maternal near-miss events in Ethiopia: a systematic review and meta-analysis. Int J Afr Nurs Sci.

[17] Haw NJL. Utilization of the Ghana National Health Insurance Scheme and its association with patient perceptions on healthcare quality. Int J Qual Heal Care. 2019;31(6). 10.1093/intqhc/mzy185 30165414

[18] Ghana Statistical Service (GSS)., Ghana Health Service (GHS) I. Ghana Maternal Health Survey 2017. Accra, Ghana: GSS, GHS, and ICF. 2018.

[19] Asante F, Chikwama C, Daniels A, Armar-klemesu M (2010). Evaluating the economic outcomes of the policy of fee exemption for maternal delivery care in Ghana. Ghana Med J.

[20] Penfold S, Harrison E, Bell J, Fitzmaurice A. Evaluation of the delivery fee exemption policy in ghana: population estimates of changes in delivery service utilization in two regions. Ghana Med J [Internet]. 2007;41(3):100–9. Available from: http://www.ncbi.nlm.nih.gov/pubmed/18470327%0Ahttp://www.pubmedcentral.nih.gov/articlerender.fcgi?artid=PMC2279083. PMC2279083 18470327

[21] Johnson FA, Frempong-Ainguah F, Padmadas SS. Two decades of maternity care fee exemption policies in Ghana: have they benefited the poor? Health Policy Plan. 2016;31(1). 10.1093/heapol/czv017 25862731

[22] Witter S, Garshong B. Something old or something new? Social health insurance in Ghana. BMC Int Health Hum Rights [Internet]. 2009;9(1):20. Available from: http://bmcinthealthhumrights.biomedcentral.com/articles/10.1186/1472-698X-9-20. 10.1186/1472-698X-9-20 PMC2739838 19715583

[23] Ansong-tornui J, Armar-klemesu M, Arhinful D, Penfold S, Hussein J (2010). Hospital based maternity care in Ghana - findings of a confidential enquiry into maternal deaths. Ghana Med J.

[24] Azaare J, Akweongo P, Aryeteey GC, Dwomoh D. Evaluating the impact of maternal health care policy on stillbirth and perinatal mortality in Ghana; a mixed method approach using two rounds of Ghana demographic and health survey data sets and qualitative design technique. PLoS One [Internet]. 2022;17(9):1–23. 10.1371/journal.pone.0274573. 10.1371/journal.pone.0274573 PMC9521900 36174023

[25] Amoro VA, Abiiro GA, Alatinga KA (2021). Bypassing primary healthcare facilities for maternal healthcare in North West Ghana: socio-economic correlates and financial implications. BMC Health Serv Res.

[26] Mugo NS, Mya KS, Raynes-Greenow C (2020). Country compliance with WHO-recommended antenatal care guidelines: equity analysis of the 2015–2016 demography and Health Survey in Myanmar. BMJ Glob Heal.

[27] Azaare J, Kolekang AS, Agyeman YN. Maternal health care policy intervention and its impact on perinatal mortality outcomes in Ghana: evidence from a quasi-experimental design. Public Health [Internet]. 2023;222:37–44. 10.1016/j.puhe.2023.06.035. 10.1016/j.puhe.2023.06.035 37515835

[28] Mosley WH, Chen LC (1984). An Analytical Framework for the Study of Child Survival in developing countries. Popul Develoment Rev.

[29] Hodgins S, D’Agostino A (2014). The quality–coverage gap in antenatal care: toward better measurement of effective coverage. Glob Heal Sci Pract.

[30] University of Ghana. State of the Nation’s Health Report. Accra; 2018.

[31] Akazili J, Welaga P, Bawah A, Achana FS, Oduro A, Awoonor-Williams JK et al. Is Ghana’s pro-poor health insurance scheme really for the poor? Evidence from Northern Ghana. BMC Health Serv Res. 2014;14. 10.1186/s12913-014-0637-7 PMC4268792 25494816

[32] Dalinjong PA, Wang AY, Homer CSE. The operations of the free maternal care policy and out of pocket payments during childbirth in rural Northern Ghana. 2017. 10.1186/s13561-017-0180-4 PMC5700011 29168019

[33] Abuosi AA, Domfeh KA, Abor JY, Nketiah-Amponsah E (2016). Health insurance and quality of care: comparing perceptions of quality between insured and uninsured patients in Ghana’s hospitals. Int J Equity Health.

