# Supplementary material for: Maternal health care utilization following the implementation of the free maternal health care policy in Ghana: analysis of Ghana demographic and health surveys 2008–2014
Source: BMC Health Serv Res. 2024 Feb 15;24:207. doi: 10.1186/s12913-024-10661-5 (PMC10870471; doi:10.1186/s12913-024-10661-5)
Supplement: Supplementary file 1 — Supplementary Material 1 [file 12913_2024_10661_MOESM1_ESM.docx]

**Supplementary figure 1. Antenatal care attendance, source: GHS report, 2016.**

**Supplementary figure 2. Skilled delivery utilization, source: GHS Report, 2016.**
